# A qualitative study exploring how young people perceive and experience substance use services in British Columbia, Canada

**DOI:** 10.1186/s13011-022-00456-4

**Published:** 2022-05-28

**Authors:** Roxanne Turuba, Anurada Amarasekera, Amanda Madeleine Howard, Violet Brockmann, Corinne Tallon, Sarah Irving, Steve Mathias, Joanna Henderson, Kirsten Marchand, Skye Barbic

**Affiliations:** 1 Foundry, 915-1045 Howe Street, Vancouver, BC V6Z 2A9 Canada; 2 Providence Health Care, 1081 Burrard Street, Vancouver, BC V6Z 1Y6 Canada; 3 Providence Research, 1190 Hornby, Vancouver, BC V6Z 2K5 Canada; 4 Faculty of Medicine, University of British Columbia, 317-2194 Health Sciences Mall, Vancouver, BC V6T 1Z3 Canada; 5 Centre for Health Evaluation Outcome Sciences, 588-1081 Burrard Street, Vancouver, BC V6Z 1Y6 Canada; 6 Centre for Addiction and Mental Health, 80 Workman Way, Toronto, ON M6J 1H4 Canada; 7 Department of Psychiatry, University of Toronto, 250 College Street, 8th floor, Toronto, ON M5T 1R8 Canada; 8 Department of Occupational Science and Occupational Therapy, University of British Columbia, 317-2194 Health Sciences Mall, Vancouver, BC V6T 1Z3 Canada

**Keywords:** Substance use, Youth, Adolescents, Young adults, Service experiences, Participatory action research, Qualitative research

## Abstract

**Background:**

Substance use among youth (ages 12–24) is troublesome given the increasing risk of harms associated. Even more so, substance use services are largely underutilized among youth, most only accessing support when in crisis. Few studies have explored young people’s help-seeking behaviours to address substance use concerns. To address this gap, this study explored how youth perceive and experience substance use services in British Columbia (BC), Canada.

**Methods:**

Participatory action research methods were used by partnering with BC youth (under the age of 30) from across the province who have lived and/or living experience of substance use to co-design the research protocol and materials. An initial focus group and interviews were held with 30 youth (ages 12–24) with lived and/or living experience of substance use, including alcohol, cannabis, and illicit substances. The discussions were audio-recorded, transcribed verbatim, and analyzed thematically using a data-driven approach.

**Results:**

Three main themes were identified and separated by phase of service interaction, starting with: *Prevention/Early intervention*, where youth described feeling unworthy of support; *Service accessibility*, where youth encountered many barriers finding relevant substance use services and information; and *Service delivery*, where youth highlighted the importance of meeting them where they are at, including supporting those who have milder treatment needs and/or do not meet the diagnosis criteria of a substance use disorder.

**Conclusions:**

Our results suggest a clear need to prioritize substance use prevention and early interventions specifically targeting youth and young adults. Youth and peers with lived and/or living experience should be involved in co-designing and co-delivering such programs to ensure their relevance and credibility among youth. The current disease model of care leaves many of the needs of this population unmet, calling for a more integrated youth-centred approach to address the multifarious concerns linked to young people’s substance use and service outcomes and experiences.

## Background

Substance use initiation is common during adolescence and young adulthood [1]. In North America, youth (defined here as aged 12–24) report the highest prevalence of substance use compared to older age groups [2, 3], alcohol being the most common (youth 15–19: 57%; youth 20–24: 83%), followed by cannabis (youth 15–19: 19%; youth 20–24: 33%), and illicit substances (youth 15–19: 4%; youth 20–24: 10%) [2]. High rates of substance use among youth are worrisome given the ample evidence linking early onset to an increased risk of developing a substance use disorder (SUD) and further mental health and psychosocial problems [4–6]. Youth are also more likely to use more heavily and in riskier ways than adults, making them especially vulnerable to substance use related harms [2, 7]. For example, polysubstance use is more common and increasing among youth [8–10], which has been associated with an increase in youth overdose hospitalizations [11]. Substance use is also associated with several leading causes of death among youth (e.g., suicide, unintentional injury, violence) [12, 13], demonstrating an urgent need to provide effective substance use services to this population.

Current evidence-based recommendations to address substance use issues among youth include a range of comprehensive services, including family-oriented treatments, behavioural therapy, harm reduction services, pharmacological treatments, and long-term recovery services [14–17]. Like with adults, these services should be tailored based on young people’s individual needs and circumstances and should consider concurrent mental health disorders which are common among youth who use substances [3, 15, 18]. Merikangas et al. [18] reported rates of co-occurring mental health disorders as high as 77% among a community sample of youth with a SUD diagnosis. Regardless of precedence, both mental health and SUD can have exacerbating effects on each other if not treated, highlighting the importance of early diagnosis and early access to care [19]. However, current practices utilizing an integrative approach to diagnose and treat SUD and concurrent mental health disorders have yet to be widely implemented [20–22]. Further, the current substance use service landscape has been largely designed to treat SUD in adult populations [17], who often require more intensive treatment compared to youth [15].

Literature suggests that there are differences between how youth and adults perceive and present substance use issues, suggesting different approaches may be needed to address substance use concerns [15]. For example, youth have shorter substance use histories and therefore often express fewer negative consequences related to their substance use, which may reduce their perceived need for services [15]. Further, the normalization of substance use among younger populations and the influence of peers and family members may also play a factor in reducing young people’s ability to recognize problems that arise due to their substance use [9, 23]. Confidentiality concerns may also prevent youth from accessing services when needed [23]. Youth are therefore unlikely to access substance use services before they are in crisis. The 2019 National Survey on Drug Use and Health [24] reported that only 7.2% of youth ages 12–25 who were identified as needing specialized substance use treatment (defined as substance use treatment received at a hospital (inpatient), rehabilitation facility (inpatient or outpatient), or a mental health centre) accessed appropriate services and that 92% of youth did not feel they needed to access specialized services for substance use. In 2020, the percentage of youth who received specialized treatment dropped to 3.6 and 98% of youth did not perceive the need for it [3], demonstrating the exacerbating effects the pandemic has had on young people’s service trajectory and experiences.

Although help-seeking behaviours to address mental health concerns among youth have been explored [25, 26], few studies have been specifically designed to explore young people’s experiences with substance use services. Existing evidence has largely focused on the experiences of street entrenched youth and youth who specifically use illicit substances (e.g., opioids, heroin, fentanyl) ([1, 27–30], (Marchand K, Fogarty O, Pellat KM, Vig K, Melnychuk J, Katan C, et al: “We need to build a better bridge”: findings from a multi-site qualitative analysis of opportunities for improving opioid treatment services for youth, Under review)), which remains an important research focus, but may not be representative of those who have milder treatment needs. As such, this qualitative study aims to understand how youth perceive and experience substance use services in British Columbia (BC) more broadly. This study also explored young people’s recommendations to improving current models of care to address substance use concerns.

## Methods

### Study design & setting

This study is part of the *Building capacity for early intervention: Increasing access to youth-centered, evidence-based substance use and addictions services in BC and Ontario* project, which aims to create youth-informed substance use training for peer support workers and other service providers working within an integrated care model. The project is being led by Foundry Central Office and the Youth Wellness Hubs Ontario (YWHO), two youth integrated health service hubs in BC and Ontario respectively. As part of this project, the BC project team conducted a qualitative research study, entitled *The Experience Project*, to support the development of substance use training. This paper focuses on this BC study, which follows standards for reporting qualitative research (SRQR) [31].

In May 2020, we applied participatory action research (PAR) methods [32, 33], by partnering with 14 youth (under the age of 30) throughout the course of the project, who had lived and/or living experience of substance use and lived in BC. Youth advisors were recruited through social media and targeted outreach (i.e., advisory councils from Indigenous-led organizations and rural and remote communities) in order to engage a diverse group of young people. A full description of our youth engagement methods has been described elsewhere (Turuba R, Irving S, Turnbull H, Howard AM, Amarasekera A, Brockmann V, et al: Practical considerations for engaging youth with lived and/or living experience of substance use as youth advisors and co-researchers, Under review). British Columbia has a population of approximately 4.6 million people, 88% of which reside within a metropolitan area; only 12% live in rural and remote communities across a vast region of land. Nationally, BC has been disproportionately impacted by the opioid crisis, counting 1782 illicit drug overdose deaths in 2021 alone, 84% of which were due to fentanyl poisoning [34]. Although more than half of BC’s population reside in the Metro Vancouver area, rates of illicit drug overdose deaths are similar across all health regions [34].

The youth partners formed a project advisory which co-created and revised the research protocol and materials. The initial focus group questions were informed by Foundry’s Clinician Working Group, based on what Foundry clinicians wanted to know about youth who use substances and how best to support them. The subsequent interview guide was developed based on the focus group learnings and debriefing sessions with the project youth advisory (see Data Collection section below). Three advisory members were also hired as youth research assistants to support further research activities including data collection, transcription, and analysis.

### Participants

Participants were defined as youth between the ages of 12–24 who had lived and/or living experience of substance use (including alcohol, cannabis, and/or illicit substance use) in their lifetime and lived in BC. Substance use service experience was not a requirement as we wanted to understand young people’s perception of services and barriers to accessing them. Youth were recruited through Foundry’s social media pages and targeted advertisements. Organizations serving youth across the province were contacted about the study and asked to share recruitment adverts with youth clients. Organizations were identified by our youth advisors and Foundry service teams from across the province in order to recruit a geographically diverse sample of youth. This included mental health services, child and family services, social services, crisis centres, youth shelters, harm reduction services, treatment centres, substance use research partners, community centres, friendship centres, schools, and youth advisories. Interested youth contacted the research coordinator (author RT) to confirm their eligibility. Youth under the age of 16 required consent from a parent or legal guardian and gave their assent in order to participate, while youth ages 16–24 consented on their own behalf. Verbal consent was obtained from participants/legal guardians over the phone or Zoom after being read the consent form, prior to the focus group/interview. A hard copy of their consent form was signed by the research coordinator and sent to the participant/legal guardian for their records.

### Data collection

Data collection began in November 2020 until April 2021. An initial semi-structured 2-h focus group with 3 youth (ages 16–24) was facilitated by 2 trained research team members, including a youth research assistant with lived/living experience. A peer support worker was also available for further support. The focus group discussion highlighted youth participants’ multifarious experiences with substance use services and the variety of substances used, which led us to change our data collection methods to individual in-depth interviews. Two interview guides were developed based on the focus group learnings to reflect the different range of service experiences. Interviews questions were reviewed and modified with the project youth advisory. Semi-structured interviews were held with 27 youth participants, which were facilitated by 1–2 members of the research team and lasted 30-min to an hour. In an effort to promote a safe and inclusive space for youth to share their experiences, participants were given the option to request a focus group/interview facilitator who identified as a person of color if preferred. The focus group/interviews began with introductions and the development of a community agreement to ensure youth felt safe to share their experiences. Participants were also sent a demographic survey to fill out prior to the focus group/interview, which was voluntary and not a requirement for participating in the qualitative focus group/interview. Due to the COVID-19 pandemic, the discussions were conducted virtually over Zoom. Participants were provided with a $30 or $50 honoraria for taking part in an interview or focus group, respectively.

### Data analysis

The focus group and interviews were audio-recorded, transcribed verbatim, and analyzed thematically using NVivo (version 12) following an inductive approach using Braun and Clarke’s six step method [35]. The research coordinator led the analysis and debriefed regularly with author KM, who has extensive experience with qualitative health research in substance use [36, 37]. The transcripts were read multiple times and initial memos were taken. A data driven approach was used to generate verbatim codes and identify themes. Meetings were also held with the youth research assistants to discuss the data and review and refine the themes to strengthen the credibility and validity of the findings, given their role as facilitators and their lived/living experience with substance use. This included selecting supporting quotes to highlight in the manuscript and conference presentations.

## Results

We interviewed a total of 30 youth participants. Socio-demographics, substance use patterns and service experiences are listed in Table 1. Participants’ median age was 21 and primarily identified as women (55.6%) and white/Caucasian (66.7%). Most youth had used multiple substances in their lifetime and over the past 12-months, with alcohol being the most common, followed by marijuana/cannabis, psychedelics, amphetamines (e.g., MDMA, ecstasy) and other stimulants, non-prescription or illicit opioids, depressants, and inhalants. More than half (55.6%) had some post-secondary education and almost all participants were either in school and/or employed (94.4%). Seventy-five percent of participants had experience accessing substance use services.

**Table 1:** Characteristics of youth participants

Characteristics	Participants ***N*** = 18^**a**^
**Socio-demographics**	*N* (%) / Median
***Age***^**b**^	
Age (Median)	21
***Gender***	
Woman	10 (55.6)
Man	6 (33.3)
Non-binary	1 (5.6)
Not sure/questioning	1 (5.6)
***Ethnicity***^**c**^	
White/Caucasian	12 (66.7)
Middle Eastern/North African	2 (11.1)
East Asian	2 (11.1)
Southeast Asian	2 (11.1)
South Asian	1 (5.6)
Chinese	1 (5.6)
First Nation/Metis/Inuit	1 (5.6)
***School or employed***	
Both	7 (38.9)
School	8 (44.4)
Employed	2 (11.1)
Neither	1 (5.6)
***Highest level of education***	
Some high school	6 (33.3)
High school diploma	2 (11.2)
Some college or technical school education	1 (5.6)
Some university	6 (33.3)
Bachelor’s degree	2 (11.1)
Master’s degree	1 (5.6)
***Living situation***	
With my parents(s) or guardian(s)	8 (44.4)
Apartment	6 (33.3)
House	1 (5.6)
Single room occupancy hotel	1 (5.6)
University dorm	1 (5.6)
Part time with parents and partner	1 (5.6)
**Substance use within lifetime (other than prescribed by a physician)**^**d**^	
Alcohol	18 (100)
Marijuana/Cannabis	18 (100)
Psychedelics/hallucinogens	12 (66.7)
Amphetamines (MDMA/ecstasy)	9 (50.0)
Stimulants (e.g., powder cocaine, crack cocaine, crystal meth, Adderall, Dexedrine, Prozac)	9 (50.0)
Non-prescription or illicit opioids (e.g., fentanyl, heroin, Percocet)	4 (22.2)
Depressants (e.g., benzodiazepines, GBH, Xanax)	6 (33.3)
Inhalants	2 (11.1)
**Substance use in the past 12-months (other than prescribed by a physician)**^**e**^	
Alcohol	18 (100)
Marijuana/Cannabis	17 (94.4)
Psychedelics/hallucinogens	8 (44.4)
Amphetamines (MDMA/ecstasy)	5 (27.8)
Stimulants (e.g., powder cocaine, crack cocaine, crystal meth, Adderall, Dexedrine, Prozac)	5 (27.8)
Non-prescription or illicit opioids (e.g., fentanyl, heroin, Percocet)	1 (5.6)
Depressants (e.g., benzodiazepines, GBH)	1 (5.6)
Inhalants	1 (5.6)
**Types of treatment for substance use (past 12-months)**^**c**^	
Counselling	11 (61.1)
Cognitive Behavioural Therapy	3 (16.7)
Dialectical Behaviour Therapy	2 (11.1)
Psychiatry	4 (22.2)
Peer support	3 (16.7)
Case management	1 (5.6)
Harm reduction services	5 (27.8)
Housing support	1 (5.6)
None (I never got treatment for substance use)	5 (27.8)
**Type of service environment accessed for substance use (past 12-months)**^**c**^	
School counsellor	5 (27.8)
Family doctor’s office	5 (27.8)
Private office or clinic	5 (27.8)
Community health centre	3 (16.7)
Community-based Integrated Youth Services (i.e., Foundry centre)	4 (22.2)
Emergency Department	2 (11.1)

Definitions: *MDMA* 3,4-Methylenedioxymethamphetamine, *GBH* gamma-hydroxybutyrate

^a^The demographic survey was voluntary. Response rate was 60% (18/30 completed)

^b^Age is missing for *n* = 1

^c^Participants could select more than 1 response. Therefore, the number of responses may be greater than the total number of participants who completed the survey

^d^Participants were asked “Which of the following substances have you used in your lifetime (other than as prescribed by a physician)?”

^e^Participants were asked “Which of the following substances have you used in the past 12-months (other than as prescribed by a physician)?”

**Fig. 1:**
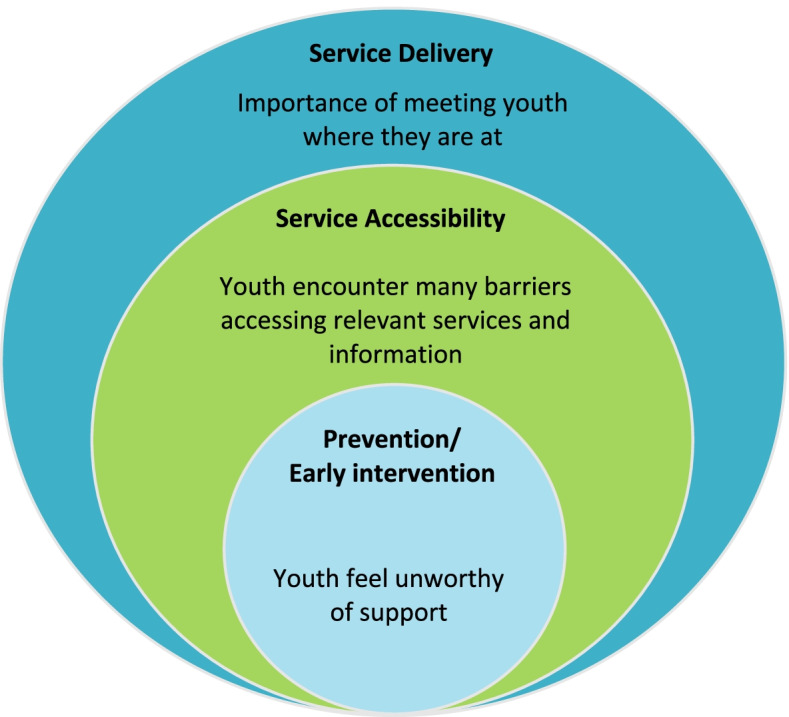
Overarching themes describing young people’s experiences with substance use services

Three overarching themes of youths’ substance use service perceptions and experiences were identified (see Fig. 1). These themes were specific to the phase of service interaction youth described, given that they were all at different phases of their substance use journeys and had different levels of interaction with substance use services. For example, some youth had never accessed substance use services but described their perceptions of services based on the information available to them, while others described specific service interactions they had. The themes were therefore separated by phase of service interaction, starting with 1. Prevention/Early intervention, where youth describe feeling unworthy of support; 2. Service accessibility, where youth encounter many barriers finding relevant services and information; and 3. Service delivery, where youth highlight the importance of meeting them where they are at.

### Prevention/early intervention: youth feel unworthy of support

Many youth described feeling unworthy of health and social services, especially when they did not identify as having a SUD. Young people’s perception of SUD typically revolved around the use of “*harder substances”,* which participants defined as heroin, crack cocaine, intravenous drugs, and being in crisis situations, such as being homeless or at risk of an overdose. Youth perceived that most services were geared towards this population and therefore not for them. Many described suffering from “*imposter syndrome*” fearing that they would be taking space away from others who needed it more or judged by services providers for accessing services they did not ‘need’:

*“...that idea that you could go get help for your drug use without it – without you being some stereotype of an addict, right?... like there’s different severities of addiction, or you could not have an addiction but also still have some sort of issue related to substance use that should be dealt with. I think my biggest fear as a person with anxiety, through all aspects of accessing health care, is that...I am gonna go to the doctor and they’re going to say ‘Oh my god what an idiot, she doesn’t need to be here, I’m just going to give her something to shut her up’.”*

Youth described feeling embarrassed or afraid of how people in the community (including friends, family, and service providers), would react to their substance use, not wanting to disappoint anyone or be stereotyped as an “*addict*”, a “*bad person*” or a “*criminal*”. Alternately, some youth were simply not ready to change their substance use behaviours and assumed this would be expected of them if they reached out for support. As one participant described: *“A lot of people are under the idea that if they tell people about their problems, they’re just going to ship them off somewhere, and the only form of recovery is abstinence based, which is not at all helpful and way too intimidating.”*

Youth also felt that substance use adverts were often irrelevant to their experiences, and that public health messaging was polarizing and unconvincing:

*“I feel like maybe there could be a larger conversation about how drugs are fun, and we should stop – like that’s the thing, if everyone pretends that they’re not and that it’s all bad – that’s why people don’t believe you, they don’t believe what you’re saying, right? Drugs are really fun, that’s why they’re dangerous. That’s why people have addiction problems. They’re really fun until they’re not.”*

*“I think if they had signs that spoke more to the average college student who is maybe getting black out every weekend or popping zanies...instead I’m hearing about a 40-year old who’s been using hard drugs for like 20 years”.*

Further, youth described how marijuana/cannabis and stimulant use were often disregarded, which are commonly used among youth and young adults [24]. For example, participants described the lack of recognition marijuana/cannabis has as being an addictive substance for some people, which invalidated their experiences. Hence, youth struggled to understand when their substance use *“hit a threshold of bad enough to bother public health services”* and therefore often only reached out for support when in crisis: *“What stopped me from accessing services after this initial attempt was me just second-guessing that I actually had an issue*”.

Youth expressed wanting more information about the neuroscience of addiction, and how to differentiate between substance use, abuse, and disorder to reduce feelings of shame and increase their ability to identify when they should reach out for support. Youth also appreciated learning that substances affect people differently, which validated their experiences*: “I learned that it’s very different for everyone....and I was like ‘Oh, I didn’t think there was anybody like me’. So it was this amazing thing, learning that I’m not the only high schooler struggling with this.”*

Youth were more likely to reach out to friends for support; however, participants reported that the normalization of substance use among youth meant peers often did not take issues seriously and therefore could not be an effective source of support long-term. This also strengthened participants’ self-doubt about whether their issues warranted support from health and social services, often delaying accessing to care.

### Service accessibility: youth encounter many barriers finding substance use services and information “zero to 100”

When youth were ready to access services and information for their substance use, they encountered many barriers. Youth expressed not knowing what services and supports were available, or which services they would benefit from: *“It seems like through my searching, it’s either you can get counselling, or you can reach out for people – to health professionals to chat with on a hotline. Or it goes from zero to 100 where you have to get admitted to a rehab treatment program.”*

Youth expressed a lack of available information about substance use and services and identified a need to reach those who were not already actively accessing services. This included advertising about different service options in schools, coffee shops, bars, and social media. *“I would’ve never went up and asked somebody about it [information about substance use services] or looked it up on the internet. That just wasn’t an interest at all.... I feel like it’s got to be in schools where you can just plain and broad see it in the office or have school counsellors talk about it.”* Youth also wanted more information provided in schools about the long-term effects of different substances, harm reduction, and how lifestyle choices and emotional regulation can play a role in substance use behaviours.

Having information more widely available was also identified to “*help break the stigma”* by increasing people’s awareness about substance use and available supports. Youth often had to research information independently, which had its own barriers. This included not knowing what to look for or where to start, a lack of information about services listed on service websites, requiring further research through phone calls and emails, and a lack of service options available. As one youth described:

*“When I saw people talking about their problems on social media...it just made me realize there’s so much other treatments out there that are just very simple. Like, you can honestly learn breathing techniques...or like cognitive behavioural therapy or all these other things...I guess for people to be able to talk about it – people don’t really see what is cognitive behavioural therapy online, you have to search it up yourself. But for some companies being able to express what it is, express what their services are, it would be able to give an idea to some people.”*

When trying to access services, youth described encountering other challenges, including long wait times, challenges getting to appointments (e.g., lack of transportation), limited hours of operation, and a lack of services available, including a lack of affordable services, especially for specialized care (e.g., service providers specializing in substance use, LGBTQ2S+, etc.). A lack of referrals between services was also a barrier to receiving care, placing the responsibility on the youth to reconnect with care, which required them to continuously retell their story. Youth also felt like service providers tended to withhold information about service options based on their level of perceived need, which was often inaccurate, and thus, felt they needed to appear more in crisis to receive more options:

*“They [service providers] will withhold certain information from you based on what your need is, because I feel like they try to assess people, and they place them on a sliding scale of like, “Who needs one more?” Which is why I didn’t really like that because … a lot of… supports only became available to me after I had been in the hospital, when I feel like I would’ve benefitted from the support even more, like beforehand.”*

### Service delivery: importance of meeting youth where they are at

For youth who accessed substance use services, their care experiences varied widely depending on their interactions with their service providers, with some who “*genuinely listened*” and “*took their time to make a connection*”, while others were described as “*uncompassionate*” and ‘*don’t really understand what I’m going through’*. Youth wanted to be “*treated with the same respect and dignity like anyone*” but described being treated like children, as though they were being “*lectured by a parent*” or treated as though incapable of making good decisions for themselves. Youth described “*not being taken seriously”* and their issues often “*pushed aside”* for not fitting a certain “*stereotype*”. For example, one participant expressed: *“I was a really good student, I had a really good home life, and everything was, on the outside, literally perfect. And there was kind of that stigma around “You don’t have any problems, why would you have problems?”.”* This strengthened youths’ perceptions that substance use services were not for them and prevented them from accessing further support. As one youth described their experience after an overdose:

*“When they had asked me my age and I had told them my age, they were like, ‘Oh my goodness. What are you doing?’ And it was just a random nurse. It wasn’t actually anyone trained, but I just felt like, ‘Wow. Maybe I should go home’. Even though I really needed to be there, it was just hard to not get up and run.”*

Youth recognized the importance of crisis-oriented services; however they expressed that *“the goal should be preventing crisis rather than just helping people when they get there.”* This implied taking youth’s concerns at face value, regardless of how service providers perceived their situation:

*“Yeah, I guess assuming that people are asking for help because they really need it, and because... people that are good at holding it together, that have extreme privilege, that look like they’re healthy and making it work, they’re still accessing services for a reason and maybe to include more of a preventative mind frame in their model of care in the sense that, this person may be not be at their worst right now, and that’s actually wonderful that they’re here before that happens, so let’s take this seriously and try to work with them before, you know, they look like they need help.”*

Having a service provider who took additional steps to support them, such as providing rides, meeting them in more casual settings, and checking in with them regularly, made youth feel genuinely cared for and increased their likelihood of returning. As one youth described:

*“I found that they checked in a lot and it made me feel like they actually cared. You know what I mean? It’s not like just because I’m not there in that moment seeing them... Sometimes, I’d get a text or a phone call being like, “Hey, what are you doing? I haven’t you seen in a while.” You know what I mean? And I had a period of time with the counsellor that I was seeing that I literally ignored her calls for 2 months and [she] was still calling me and leaving voice mails. Even though I wasn’t answering and speaking to her, I still felt like, "Wow, she actually gives a shit. She's still trying to communicate and be there even though I’m not putting the same effort back.”*

Being able to connect with someone of similar age, gender, and race/ethnicity generally made it easier for youth to relate to their service provider, however this varied and highlighted the importance of providing youth with options to choose from. Youth described being more comfortable talking to someone who could relate to them and had their own lived experiences. Hearing about similar experiences helped youth feel “*normal*” and validated. This came in the form of peer support, friends, support groups, and online forums such as Reddit and Facebook groups. However, some youth described hesitancy accessing peer support services given that peers may not have received any formal substance use training. Meanwhile, some youth assumed their problems would not compare to the lived experiences of peer support workers, and therefore did not see its value. As one youth described *“Hearing [about] other people’s problems...[it] reminds me that other people have gone through wars and made it out of wars, which is like, would be comforting for some people, but for me, makes me feel like [I should] “get over it”.”*

Youth desired a holistic approach to care, where all aspects of their life were considered rather than solely focusing on their substance use. As one participant describes: *“It wasn’t just substance abuse going on for me, so programs kind of like CBT again, it kind of helps you deal with emotions no matter what way you choose to cope...I think just more effort to get to the root of the problem instead of just trying to stop the symptom.”* Focusing on accomplishments rather than abstinence was important, as abstinence was not always young people’s objective for accessing services. Setting more attainable and flexible goals also reduced pressures associated with potential relapses, which were often a source of shame. Having providers who rejected the “*all or nothing approach*” made youth feel more confident and comfortable admitting setbacks.

Addressing mental health concerns was also a priority for most youth, many for whom it had been the primary reason for their service visit. *“When I started talking about my mental health as a factor in substance abuse rather than two different things...once I figured out what works for me...and that [mental health] was more stable, everything fell into place after that.”* Other factors youth wanted service providers to consider included traumatic experiences, parental substance use, school and work stress, social pressures, and relationship issues. Youth also found it helpful when service providers helped them build recovery capital, including helping them meet their basic needs, recommending school and employment programs, and finding activities and healthy habits. As one youth described *“We talked about lots of different ways to cope and things that do not necessarily have anything to do with my substance use, such as eating habits and exercising and study habits when I’m in school. Those really impact me. When those are going well, then it is easier for me to heal from my substance use.”*

## Discussion

Youth experience many challenges engaging with existing substance use services in BC as they are currently delivered. Participants in our study described their perceptions towards substance use and their experiences trying to navigate services, and they reflected on multi-level barriers associated with accessing information and support. Throughout these discussions, youth described how the crisis-oriented state of the current health care system leaves many of their needs unmet, calling for a more youth-centred and driven preventative and early intervention approach for diverse youth across BC.

In accordance with the Canadian Drugs and Substances Strategy [38], all three themes demonstrate a clear need to prioritize substance use prevention and early intervention specifically targeting youth. Youth are in the early phase of substance use, which presents a critical opportunity to reduce potential related harms, including SUDs. However, many existing prevention programs and early interventions have shown limited effectiveness in reducing substance use and associated harms among youth [39], and very few youths receive evidence-based substance use prevention and education [40, 41]. Hanley et al. [41] reported only 35% of schools in the United States used evidence-based programing, and that only 14% used evidence-based strategies as their primary source of programming. Programs like D.A.R.E. are still being used [42], which focus on the potential negative consequences associated with substance use to deter young people from using, rather than acknowledging their place in society [43, 44]. This approach fails to acknowledge that youth often use substances for enjoyment and social benefits, rather than solely responding to distress [44, 45], leading to unconvincing public health messages that fail to resonate with youth.

Following the principles of the Canadian Standards for Community-Based Youth Substance Abuse Prevention [46], substance use prevention and education should be informed by youth to ensure messaging is relevant to their experiences and is effective in providing youth with the tools needed to make informed decisions about substance use. Moffat et al. [47] reported that involving youth in prevention efforts helped develop public health recommendations about cannabis that were less ambiguous and stimulated productive conversations among youth about the associated risks. A systematic review on the involvement of youth in substance use prevention efforts also reported that these practices increased youths’ knowledge about substance use and supported the development of prevention interventions that were specifically tailored to the needs of the community [48].

Youth participants also highlighted the benefits of hearing from peer experiences and advocated for more opportunities for peers to talk in schools. Although there has been increasing evidence supporting the effectiveness of peer-led programs in reducing substance use and associated harms, peers remain largely underutilized in substance use prevention efforts [49, 50]. These findings underline the importance of reducing stigma and discrimination against people who use substances, so that peers can be actively engaged in programs design and delivery. However, the findings from this study also indicates that youth may worry about peers invalidating their own experiences through self-disclosure, highlighting the different preferences among youth. This also suggests that the purpose of self-disclosure may need to be better conveyed to youth as a tool to help build common humanity and trust rather than the focus of peer roles.

The study also highlighted that preventative efforts are not only important in school settings but should also be applied in other healthcare settings. As youth from this study explained, services should address the motivations for using substances from a holistic perspective rather than trying to treat substance use alone, requiring an individualized approach. Concurrent mental health disorders, including internalizing (e.g., anxiety, depression) and externalizing disorders (e.g., attention deficit hyperactivity disorder, conduct disorder) are common among youth and are often linked to substance use issues, highlighting the importance of diagnosing and treating substance use and mental health concerns simultaneously [22, 51]. However, our results emphasized that the current fragmented state of the healthcare system makes this approach challenging for young people and their families. As many youths access the healthcare system for reasons other than substance use concerns, substance use screening and brief interventions need to occur in a variety of health care settings, accompanied with proper staff training. This approach has been proven to be effective in reducing substance use and violence among youth by screening for substance use in schools, emergency departments, and primary care settings among high-risk youth [52]. However, this study suggests that substance use screening should be applied more broadly and intentionally integrated as youth may not present external signs of problematic substance use and may not feel comfortable bringing it up unless explicitly asked or in crisis. Providing service providers with training on how to provide culturally safe care to youth who use substances is imperative for this approach to be effective and maintain trusting relationships with youth, given young people’s fears of being stigmatized and judged when accessing services [53, 54].

There has been increasing evidence supporting the benefits of an integrated approach to address substance use and mental health concerns among youth, which would facilitate the early identification of possible substance use issues [21]. Although several barriers can impede the implementation of such services (e.g., organizational-level barriers, distinct health financing systems, and having to train providers in multiple disciplines) [54], this model of care has been successfully implemented in Australia (Headspace) [55], Ireland (Jigsaw) [56], and Canada (Foundry, Youth Wellness Hubs Ontario, ACCESS Open Minds, and YouthCAN Impact) [21, 57]. This framework has the potential to increase service provider awareness about the complexities associated with substance use and facilitate the delivery of a wide range of services to support recovery, such as primary care, financial assistance, supportive housing, employment, education, and family support. Given youths’ hesitancy to discuss substance use issues with health care providers, this framework should also integrate peer support services to provide youth with a relatable point of contact to discuss issues without fear of judgment or negative consequences [21]. Although peer support has been associated with positive treatment outcomes [58], this study suggests that these services need to be better integrated and conveyed to youth who may benefit.

The service accessibility barriers described by youth in this study reflect the undeniable need to increase the service system’s capacity to provide substance use services. These barriers are consistent with other Canadian studies [26, 59, 60], including a study conducted with youth in urban, rural, and remote Ontario [59] who described a general lack of substance use services available, low service awareness by youth, and a lack of coordination and collaboration between services. Family members in this study validated these challenges as they described trying to navigate the system for and/or with their young person, which was further substantiated by caregivers trying to navigate youth opioid treatment services in BC (Marchand KM, Turuba R, Katan C, Brasset C, Fogarty O, Tallon C, et al: Becoming our young people’s case managers:Caregivers’ experiences, needs, and ideas for improving opioid use treatments for young people using opioids, Under review). Given the increasing harms associated with the opioid crisis [7], coordinated efforts across all levels of government and multiple sectors are imperative to improving young people’s access to substance use services and create space, not only for youth in dire need of these services, but for those trying to address substance use concerns proactively.

This study had several limitations. Participants were recruited through Foundry social media channels and targeted advertisements, therefore youth who had access to a phone or a computer and followed mental health and/or substance use organizations were more likely to hear about the study. Consequently, our sample mainly included youth who were actively employed and in school and living in stable living environments. Yet, similar accessibility barriers are described by street-entrenched youth in Ontario [27] and British Columbia [30], including long wait times and difficulties seeking support due to stigma, as well as negative experiences with abstinent-based approaches, highlighting young people’s desire for holistic care regardless of substance use patterns. Although we tried to recruit through several health and social services across the province, the COVID-19 pandemic likely limited organizations’ capacity to support with local promotion. Further, we were only able to recruit 1 youth between the ages of 12–15, likely due to our inability to recruit through schools and need for parental consent, which hindered our ability to identify potential differences in substance use service perceptions and experiences between adolescents and young adults. Given the important life transitions that occur between adolescence and young adulthood, future studies exploring these differences are important as different prevention and early intervention approaches may be warranted. Exploring how perceptions and experiences differ across communities could also be an important consideration for future research to better understand how geographic location, including urban and rural differences, impacts young peoples’ access to services. Despite these limitations, the findings of this study have important implications in the way we co-design and deliver substance use services to youth. They also have important considerations for policy makers who are considering how to shape substance use services for diverse youth in their jurisdictions.

## Conclusions

This study highlights the many challenges youth experience when engaging with substance use services and emphasizes a need for a more preventative approach. The lack of integration and capacity among service providers to provide substance use services implies that youth who have milder treatment needs and/or do not meet the diagnosis criteria of SUD often do not have access to adequate substance use service interventions. Research, health service, and policy efforts should focus on substance use prevention and early interventions to address young people’s concerns before they are in crisis and increase their ability to perceive the need to reach out for support. Moving forward, it is critical that diverse youth and peers with lived and/or living experience be involved in these efforts, including the co-design of new services and evaluation of impact of prevention and early intervention services, including quality improvement efforts. Intentional, sustained investment in youth substance use services will optimize the health outcomes and experiences of young people across BC, transformation that young people can no longer patiently wait for.

## Data Availability

The datasets generated and analysed during the current study are not publicly available due to the potential for identifying participants but are available from the corresponding author on reasonable requests.

## Abbreviations

BC: British Columbia
D.A.R.E.: Drug Abuse Resistance Education
MDMA: 3,4-Methylenedioxymethamphetamine
PAR: Participatory action research
SUD: Substance use disorder
